# The Use of Artificial Intelligence Approaches for Performance Improvement of Low-Cost Integrated Navigation Systems

**DOI:** 10.3390/s23136127

**Published:** 2023-07-03

**Authors:** Giorgio de Alteriis, Davide Ruggiero, Francesco Del Prete, Claudia Conte, Enzo Caputo, Verdiana Bottino, Filippo Carone Fabiani, Domenico Accardo, Rosario Schiano Lo Moriello

**Affiliations:** 1 Department of Industrial Engineering, University of Naples Federico II, Piazzale Tecchio 80, 80125 Naples, Italy; claudia.conte2@unina.it (C.C.); enzo.caputo@unina.it (E.C.); verdiana.bottino@unina.it (V.B.); domenico.accardo@unina.it (D.A.); rschiano@unina.it (R.S.L.M.); 2 STMicroelectronics, Analog, MEMS and Sensor Group R&D, 80022 Arzano, Italy; davide.ruggiero@st.com (D.R.); francesco.delprete@st.com (F.D.P.); 3 Department of Economics, Management and Statistics, University Milano-Bicocca, 20126 Milano, Italy; filippo.caronefabiani@unimib.it

**Keywords:** artificial intelligence, neural network, MEMS, Kalman filter, redundant-IMU

## Abstract

In this paper, the authors investigate the possibility of applying artificial intelligence algorithms to the outputs of a low-cost Kalman filter-based navigation solution in order to achieve performance similar to that of high-end MEMS inertial sensors. To further improve the results of the prototype and simultaneously lighten filter requirements, different AI models are compared in this paper to determine their performance in terms of complexity and accuracy. By overcoming some known limitations (e.g., sensitivity on the dimension of input data from inertial sensors) and starting from Kalman filter applications (whose raw noise parameter estimates were obtained from a simple analysis of sensor specifications), such a solution presents an intermediate behavior compared to the current state of the art. It allows the exploitation of the power of AI models. Different Neural Network models have been taken into account and compared in terms of measurement accuracy and a number of model parameters; in particular, Dense, 1-Dimension Convolutional, and Long Short Term Memory Neural networks. As can be excepted, the higher the NN complexity, the higher the measurement accuracy; the models’ performance has been assessed by means of the root-mean-square error (*RMSE*) between the target and predicted values of all the navigation parameters.

## 1. Introduction

Accurate measurements of the state of motion of a vehicle or object turn out to be a key aspect of several fields peculiar to the current industrial revolution [1]. These range from autonomous navigation systems of air and ground vehicles for smart city applications, to the autonomous and fast transportation of goods and products within warehouses, and from the control of robotic arms to the positioning and monitoring of large building structures [2,3,4,5,6,7]. For each of these applications, knowledge of kinetic parameters consisting of position, velocity, and attitude is required in order to determine any maneuvers and/or activities necessary to achieve the final task. Several solutions are available in the literature and on the market, which differ in hardware costs, the complexity of processing procedures, and quality of estimation—characteristics that are, as is often the case, antithetical to each other [8].

Concerning hardware components, the main cost item is the inertial measurement unit (IMU); in particular, their performance is classified according to the so-called degree of operations [9,10,11,12]. Navigation grade systems guarantee the best performance based on fiber-optic sensors, which are bulky and expensive, but their value of typical errors (bias, drift, misalignment, and scale factor) is as low as to guarantee satisfactory inertial navigation, even without the need for further corrective work. The quality of IMU systems then decreases until they reach the other end of the scale, the consumer-grade systems, typically used for gaming or automotive applications [13,14,15,16]. Typically, these sensors are made of MEMS technology, which achieves significant improvements in size, weight, and cost [17]. 

Their geometric and physical characteristics make them ideal for applications involving small autonomous vehicles, to the detriment of worst metrological performance. Unlike high-end solutions, the outputs of low-level MEMS sensors must be processed by appropriate numerical signal processing algorithms to enable them to be used in navigation applications [18,19,20]. For this purpose, the information provided by other sensors (GNSS, radar, optical sensors, odometers, etc.) is usually used to go in, estimate, and subsequently compensate for the negative effects of bias and other sources of uncertainty. The resulting system constitutes an Integrated Navigation System (INS) and typically exploits the Kalman filter for integrating measurements from inertial sensors and external sensors [21,22]. 

Several versions of the Kalman filter have been proposed in the recent past in order to improve the performance of the navigation system; in fact, in its original version, the Kalman filter was proposed for state estimation of linear systems through a prediction/correction type approach. Unfortunately, the equations describing the motion of vehicles and objects turn out to be nonlinear and, as a result, make the direct application of Kalman filters impossible or incorrect in the long run; solutions based on Extended Kalman filters (EKF) or Unscented Kalman filters (UKF) have been proposed to overcome this limitation [23,24].

The first approach is based on linearizing the nonlinear system by expanding the model functions in truncated Taylor series to first order; the EKF and its subsequent modifications are among the most commonly exploited solutions not only in navigation but throughout engineering. Unfortunately, when the model equations have a very high degree of nonlinearity, the quality of the EKF estimates becomes poor. The second approach attempts to provide a solution to this problem by assuming that it is easier to approximate a probability distribution than a nonlinear function. In this way, it is also possible to carry higher-order terms within the filter computations, making the state estimates closer to their true values [25,26,27,28].

Although capable of satisfactory results for traditional applications of estimating the navigation and motion state of vehicles and objects, such solutions are sensitive to the value provided to the noise matrices used in their implementations; any unsuitable choices of such parameters lead to sub-optimal solutions that can be improved only through patient tuning of the values of the matrices [21].

To overcome these limitations, several solutions have recently been proposed that take advantage of modern artificial intelligence (AI) models to improve the performance of inertial sensors; a very comprehensive and organized survey of several solutions is presented in [29] in which advantages and shortcomings of such solutions are analyzed in detail. In particular, such algorithms are applied to the outputs of inertial sensors to directly obtain navigation state parameters. The main problems related to AI algorithms concern the amount and the quality of the data for their training and assessment and, mostly, the leak of grip on physical and geometric aspects of the real problem.

Aware of these limitations and supported by our previous study on a redundant inertial sensor system, here we want to discuss the possibility of applying AI algorithms to the outputs of an integrated navigation algorithm in such a way as to ensure performance close to that of high-end MEMS inertial sensors (tactical grade costing about EUR 5000). In particular, different AI models are compared in order to determine their performance in terms of complexity and accuracy. Such a solution presents an intermediate behavior with respect to state of the art summarized above, allowing the exploitation of the benefit of AI models by overcoming their highlighted limitations (the inputs are quantities that have already been processed by means of the geometric equations of motion) and starting from Kalman filter applications whose noise parameters were obtained from a simple analysis of sensor specifications.

The paper is organized as follows; the authors’ past experience and state-of-the-art are presented in Section 2, the proposed method and implementation of the AI-based navigation solution are described in Section 3, while in Section 4, the obtained results are presented as advantages introduced by the proposed AI approach and the overall performance reached, before drawing the conclusions in Section 5.

## 2. Related Work

To better appreciate the improvement brought by the present paper, a brief literature state-of-the-art is presented in the next section. In particular, Section 2.1 describes the hardware and software architecture of an IMU prototype based on a redundant configuration of cost-effective inertial sensors; the prototype has been presented in [30], and suitable results have been achieved thanks to an accurate estimation of the noise parameter of the adopted Kalman filter. On the contrary, the main proposals based on AI models are summarized in Section 2.2, highlighting benefits and limitations.

### 2.1. Realized Prototype of a Redundant Inertial Measurement Unit

The well-known benefit of the adoption of low-cost MEMS sensors has led the author to evaluate methods, both hardware and software, to adopt this category of sensors also in application fields such as aerospace, where the performance requirements represent a critical aspect. To this aim, a redundant configuration of six inertial sensors, both accelerometers and gyroscopes, was developed. In fact, it was proved that the IMU bias uncertainty could be reduced by exploiting a geometrical redundancy [30].

The realized prototype was composed of six IMU referred to as SensorTile^TM^ from STMicroelectronics (Geneva, Switzerland); each SensorTile includes 13.5 × 13.5 mm^2^, a low-cost inertial sensor (iNEMO), eCompass module, barometric pressure sensor, digital MEMS microphone and Bluetooth low energy module that are managed by a 32-bit ultra-low-power Cortex-M4 80-MHz microcontroller. The sensor boards are then connected with a microcontroller, called STM32F303K8 from STMicroelectronics, that acts as a concentrator, i.e., collect the raw acceleration and angular velocity measurements from the IMUs by means of the Serial Peripherical Interface (SPI) protocol, and can send the acquired data through the UART protocol or can be stored in a micro-Sd card. The prototype was configured in such a way that the inertial measurements are acquired with a frequency of about 125 Hz, exploiting the SPI protocol communication speed capabilities where the frequency was set to 10 MHz. Moreover, a GNSS module from STMicroelectronics^TM^, Geneva, Switzerland (X-Nucleo GNSS1A1) is placed on a SensorTile board that is connected to the microcontroller by means of UART communication.

To verify the prototype performance, the results obtained from the prototype were compared with a tactical-grade IMU, called STIM300 from Sensonor, that is selected as a reference system. In fact, this compact IMU presents a tri-axial accelerometer and gyroscope with remarkable performance; in particular, the gyroscope angular Random Walk (RW) and Bias Instability (BI) are equal to 0.15 deg/$\sqrt{h}$ and 0.3 deg/h, respectively, while the accelerometer velocity random walk and bias instability are equal to 0.07 m/s/$\sqrt{h}$ and 0.04 mg, respectively. These performance values allow the adoption of these sensors in aerospace applications, but their cost is five orders of magnitude higher than the commercial-grade mems.

As already introduced, the inertial sensors need the integration of an external source that provides information about the position and velocity to integrate them with the inertial measures by means of a data fusion algorithm. In fact, in this research, a loosely coupled GNSS/INS Kalman Filter-based architecture has been presented. In particular, the same GNSS module for the prototype and the reference system is selected, called Teseo-LIV3, from STMicroelectronics^TM^, Geneva, Switzerland.

**Figure 1:**
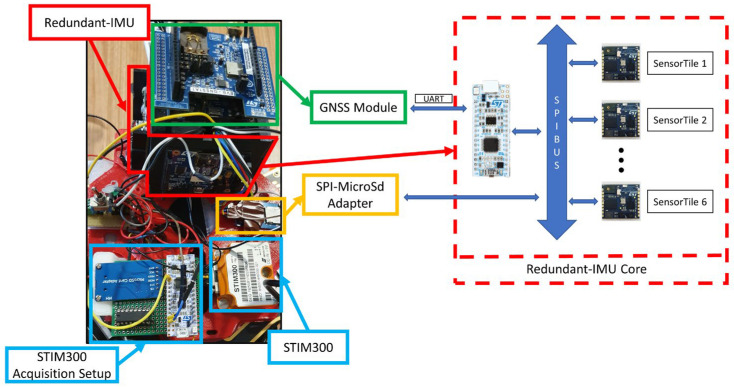
Hardware architecture composed of a redundant imu (in red), a GNSS module (in green), a SPI-SDcard adapter (in orange), and the STIM300 (in blue).

To better summarize the hardware architecture proposed, a full components and realization scheme is proposed in Figure 1.

On the other hand, a calibration procedure that resolves the relative alignment has been evaluated to ensure comparable performance with the reference system and exploit the redundant configuration benefits. In particular, by evaluating the gravity vector on each IMU in six different orientations, it was possible to obtain the transformation matrix for each reference frame; in such a way, the relative alignment of each IMU is referred to as a single reference frame. This procedure resolves not only the different reference frames but also the residual misalignment of each IMU. Once obtained the transformation matrix as a one-time calibration procedure, the prototype errors, such as bias instability and random walk, are evaluated according to the IEEE standard [31], which involves the acquisition of acceleration and angular velocity measurements for a time equal to 48 h at a constant temperature. In fact, by means of the Allan variance, the error parameters could be evaluated from the curve portion according to the IEEE standard. These parameters are needed to configure the Kalman filter noise covariance matrix properly.

Finally, after the one-time prototype calibration and characterization, two Kalman filter-based algorithms have been developed. The first one, called Zero-Velocity Update (ZUPT) filter, is adopted to initialize the bias values used in the GNSS/INS navigation filter; it consists of the position and attitude estimate in stationary conditions where the residual estimated errors are evaluated as sensors bias. As evaluated in this research, the bias initialization procedure achieves high performance in only 60 s. The second one is applied to estimate the attitude and position in dynamic conditions. Actually, the state vector is initialized with 15-state that are the attitude, position, velocity errors, and bias values (along the three axes of the accelerometers and gyroscopes).

**Figure 2:**
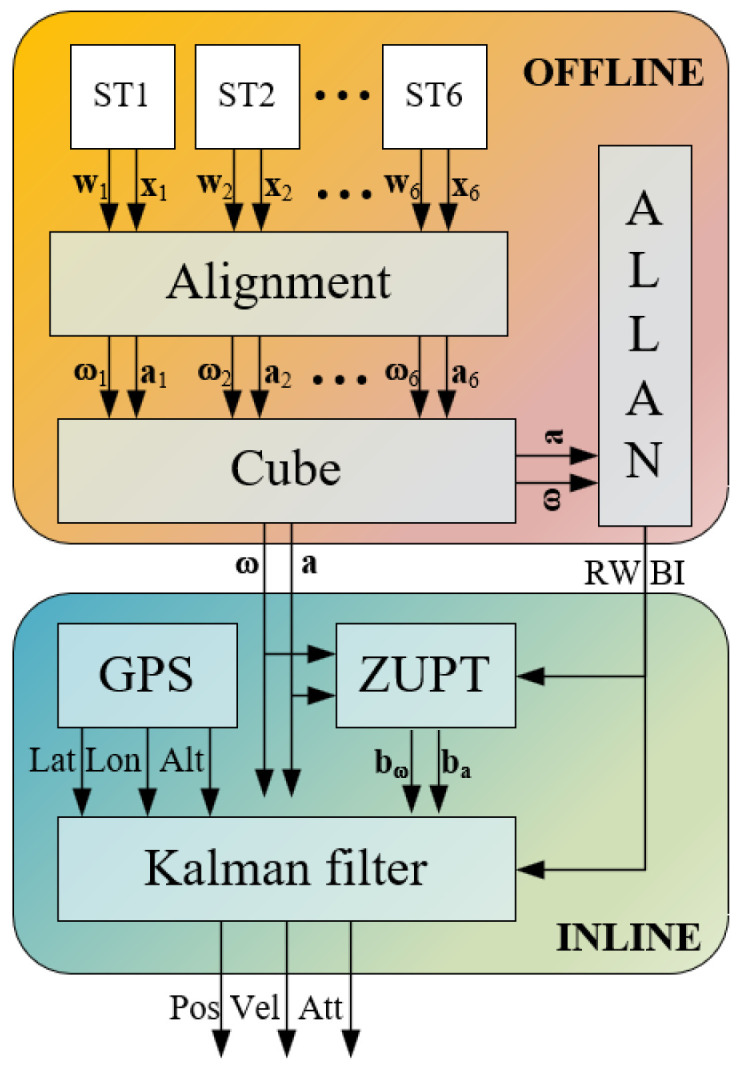
Proposed method for navigation parameter estimates.

For the sake of clarity, the proposed method is shown in Figure 2, where the acceleration (*a*) and angular velocity (*w*) measurements are collected from the six SensorTile (ST). Successively, they are aligned according to the procedure described in [30], and the noise parameters, i.e., RW and BI, are evaluated by means of the Allan Variance. In the navigation phase, preliminary initial bias estimations, that are, the accelerometer (*b_a_*) and gyroscope (*b_w_*) bias values, have been realized with a ZUPT filter, and then the position, velocity, and attitude values are estimated by means of an integrated navigation filter, i.e., loosely-coupled Error-State Kalman Filter that process the inertial measurements and the GNSS data.

### 2.2. Artificial Intelligence for Inertial Sensing

As for all the scientific and industrial fields and applications, also in inertial and integrated navigation, several papers have been presented in order to investigate the advantages brought by the exploitation of AI techniques [29]. In particular, Machine Learning (ML) has been adopted to enhance inertial sensor performances at different stages of their typical application fields, from a fundamental hardware level (e.g., gyros lifecycle estimation [32]) to calibration and error modeling (e.g., ANN for thermal drift compensation [33]), from inertial navigation (e.g., a machine-learning algorithm for Euler angle measurements [34]) to high-level applications (e.g., action classification based on IMU by means of ANN [35]).

Hereafter, the attention will be focused on the application of machine-learning algorithms to enhance the performance of integrated navigation solutions based on multi-sensor information fusion. As an example, different approaches (either regression models or classification algorithms) have been compared in [36] to detect the sideslip of the robot. Even though machine-learning approaches were characterized by the same accuracy in sideslip detection of the classification algorithms, they proved worst from a computational burden point of view, which is relevant for this kind of application. Authors in [25] exploited the hidden Markov process to estimate the presence of electromagnetic interference and, consequently, suitably weight the correction effect of the magnetic field in an attitude and heading reference system, thus enhancing its performance. Unfortunately, the authors only assessed the performance improvement due to the HMP without comparing other solutions available in the literature. Some solutions are mandated to improve sensors measurements for the successive exploitation in the information fusion filter (as an example, [37]); as can be expected, the greater the number of sensors, the greater the computational burden of the approach, thus inherently limiting the scalability of the method.

Several papers have been proposed where different machine-learning algorithms are exploited to realize the data fusion of various sensors with the data coming from the inertial sensors, mainly GNSSs, cameras, odometers, and magnetometers [38,39,40] or ensure the performance maintenance of the navigation system if the external correction information would not be available [41]. As an example, the enhancement brought by an LSTM ANN to an inertial navigation system for visual odometry applications is discussed in [39], where the associated improvements are clearly presented from a quantitative point of view but not compared to other possible geometric-based approaches. On the contrary, ML is exploited in [41] to realize a tightly coupled GNSS receiver by predicting the raw measurements from external sensors rather than the corrections they produce in the information fusion filter.

The main drawbacks of the latter approaches, with respect to other solutions proposed in the literature, can be found in the lack of either the performance or the analysis capability of the navigation quality. To overcome the above limitations, the authors present hereinafter the comparison of different machine-learning solutions in order to investigate and assess their performance from both accuracy and computational burden points of view. Differently from the solutions considered so far, the proposed models are applied to the outputs (position, velocity, and attitude) provided by a sub-optimal information fusion filter, whose noise parameters have not been properly tailored for the application [30] but are roughly estimated starting from the poor data sheet data of the inertial sensors.

## 3. Proposed ANN-Based Navigation Solutions

**Figure 3:**
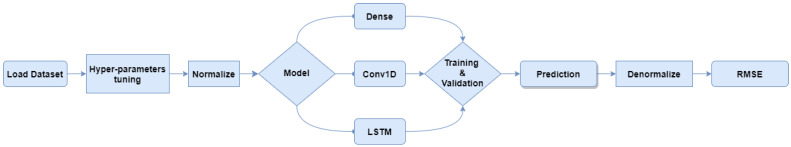
Block diagram of the proposed ML-based navigation solution.

As stated above, the paper aims to investigate the suitability of machine-learning algorithms to improve the performance of a loosely coupled integrated navigation system based on a redundant configuration of low-cost, consumer-grade MEMS inertial sensors. To this aim, ML models are trained in order to provide estimates of the navigation parameters as close as possible to those assured by high-end, tactical-grade MEMS sensors. The operating steps of the method are summarized in the block diagram of Figure 3 and mainly enlist:

- Data preprocessing;
- Neural Networks (NN) models developing;
- Model’s training and validation;
- Hyper-parameter tuning.

**Figure 4:**
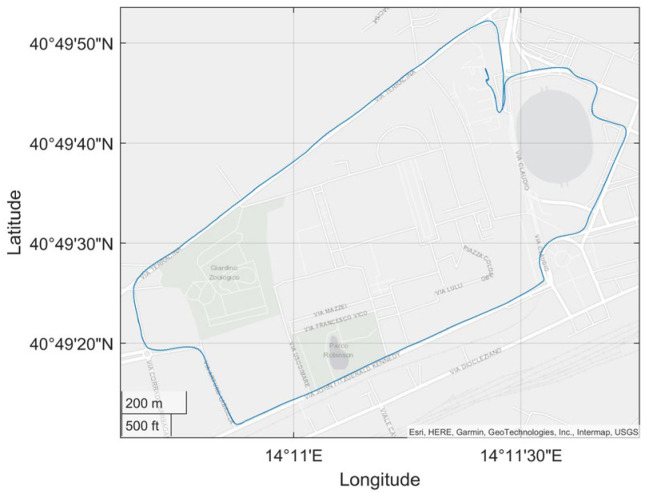
Actual car route exploited as an application example for the presentation of the operating steps of the proposed ML-based navigation solution.

Details of the considered steps are given in the following subsections. For the sake of clarity, the proposed method will be presented by considering an application example involving an actual car ride whose track is shown in Figure 4.

**Figure 5:**
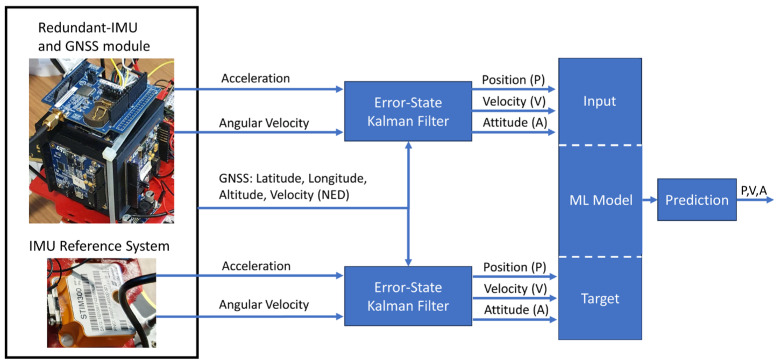
Machine Learning solution and navigation parameter estimations based on an Error-State Kalman Filter.

In this application, the inertial data are acquired from the STIM300 and Cube at 125 Hz while the position and velocity (in NED reference frame) are acquired from the GNSS module with lower frequency (1 Hz), then are processed by means of an Error-State Kalman Filter (ESKF). Both estimates obtained from ESKF, i.e., latitude, longitude, altitude, attitude (heading, pitch, and roll), and velocity (in NED reference frame), are then taken into account; in particular, the estimates obtained from the STIM300/GNSS are considered as reference (Target) while those obtained from Cube/GNSS (Input) are processed by means of ML algorithm as shown in Figure 5.

### 3.1. Data Preprocessing

The input dataset for the ML models consists of the output of the redundant configuration of SensorTiles^TM^ (the Cube) integrated with a GNSS Teseo LIV3F by a Kaman filter whose noise parameters were not optimized as in [30] but obtained from datasheet information. In this way, the estimates of navigation parameters are poorer with respect to those provided in [30], but no specific optimization (as an example, Allan variance) is required, with a consequent gain in terms of implementation time.

Actually, the dataset includes about 81,000 samples with 9 parameters that are estimates for both the Cube and the STIM integrated with GNSS, i.e., altitude, latitude, longitude, heading, pitch, roll, and velocity along the three axes; the main statistical parameters of the dataset are summarized in Table 1.

As for the ML model definition and training, the navigation parameters that are estimated by means of the Cube/GNSS integration represent the input for the NNs, while those provided by the STIM/GNSS integration represent the corresponding target. Before being processed by the proposed models, the dataset is first preprocessed to improve the training success rate of the ANN. More specifically, the preprocessing steps are:

1. Normalizing the dataset;
2. Removing outliers with the z-score technique;
3. Split the dataset into two parts: train and test.

The data normalization step transforms the parameter values by scaling each of them within the interval between 0 and 1; to this aim, the Python Scikit-Learn MinMaxScaler [42] estimator has been exploited to individually scale each parameter from its original range in the dataset to a new interval in order to speed up the training phase of the NN. The data normalization was conducted, paying attention to avoid data leakage from the test dataset to the training dataset.

The standard score technique (usually referred to as z-scores or standard-score [43]), exploited for the outlier’s removal step, is calculated by subtracting the mean value of processed data from each raw observation and then dividing the difference by its standard deviation. Values of the parameters characterized by a standard score greater than three have been removed.

To estimate and validate the performance of the NN, the Train-Test split procedure has been used where the dataset was split according to the following standard percentages: train 80% and test 20%, i.e., the final 20% has been exploited for the testing stage (acting this way also as validation).

In the training phase, the NNs models receive the inputs (Cube outputs processed by the Kalman filter), generate the output (predictions) and compare the latter with the target data (STIM outputs processed by the Kalman filter).

A crucial step in the design phase of a NN is the choice of model hyperparameters, where a hyperparameter means all the parameters which control the learning process of the model, unlike the network weights, which are identified as the parameters of a NN architecture.

A brief description of the hyperparameters involved in our NNs will now be given:

- In a NN, the loss function quantifies the difference between the expected outcome and the outcome produced by the model. From the loss function, we can derive the gradients which are used to update the weights. The average overall losses constitute the cost. A loss function based on the *MAE* was used for all the developed models;
- To assess the prediction performance of the models, two performance factors (*MAE* and *RMSE*) have been taken into account to the purpose, i.e., (i) the concurrence between estimated and nominal navigation parameters, and (ii) the number of parameters to be determined and trained for the ANN model.

The Mean Absolute Error (*MAE*) and *RMSE* are expressed, respectively, as:

(1) $$MAE=\overset{N-1}{\underset{i=0}{\sum }}\frac{\left|x_{T}\left[i\right]-x\left[i\right]\right|}{N}$$

(2) $$RMSE=\sqrt{\overset{N-1}{\underset{i=0}{\sum }}\frac{{\left(x_{T}\left[i\right]-x\left[i\right]\right)}^{2}}{N}}$$

where *x*[*i*] stands for the i-th value of a generic navigation parameter of the Cube (either original or predicted by the ANN model), $x_{T}\left[i\right]$ is the corresponding value provided by the STIM navigation, and N is the number of considered samples.

- An optimization algorithm (optimizer) finds the value of the parameters (weights) that minimize the error when mapping inputs to outputs. These optimizers widely increase the accuracy and speed training of the model as well. In the design of our NNs, we selected Adam [44] as the optimizer. Adam is an alternative optimization algorithm that provides more efficient weights by running repeated cycles of “adaptive moment estimation.” Adam extends on stochastic gradient descent to solve non-convex problems faster while using fewer resources than many other optimization programs;
- The learning rate is a tuning parameter of the optimization algorithm that controls the update of the network weights, moving towards the minimum of the loss function. Choosing the learning rate is challenging in that a too-small value may imply a time-consuming training process, whereas a too-large value may result in achieving learning a sub-optimal set of weights too fast since an unstable training process;
- The batch size is a hyperparameter of gradient descent-based optimizers that control the number of training samples to work through before the model’s internal parameters are updated;
- The number of epochs is a hyperparameter of gradient descent-based optimizers that control the number of complete passes through the training dataset.

During the training process, the weights of the NN, starting from a random initial condition, are optimized to reduce the model prediction error. In the testing phase, the network generates its outputs (predictions) in an unsupervised manner, and the concurrence with the target data is considered. Different hyperparameter values have been chosen for our models, and the relative values will be shown in the next paragraphs; actually, three different models of NN have been compared to improve the navigation performance of the low-cost redundant IMU configuration.

### 3.2. Dense Neural Network Model

The first model taken into account is a Dense Neural Network (DNN) [45] characterized by an input layer with 335 neurons, two hidden layers with, respectively, 479 and 579 neurons, and an output layer. In a DNN model, a layer is fully connected with its preceding layer; each neuron of the layer is connected to every neuron of its preceding layer; hence it receives outputs from every neuron of its preceding layer.

**Figure 6:**
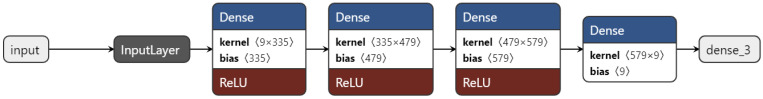
Architecture of the adopted dense neural network model.

As shown in Figure 6, the activation function used for the neurons of each layer is the ReLU [46], but the last one has a linear activation function since the task addressed is a regression task. The activation function φ is the decision-making element that defines the decision boundary in the input space by setting a threshold.

In particular, Figure 6 shows the feed-forward layer of the DNN model. The training phase of the network takes place in the backpropagation layer [47]. In this layer, the chosen optimization algorithm, Adam, tunes the network parameters (weights). The hyperparameter values chosen for this model are shown in Table 2, along with a brief description of the network architecture in terms of layers distribution and the number of corresponding parameters.

This model is characterized by 447,434 weights, each of which can be trained. The trainable parameters associated with each neuron are the weights (w) and the biases (b). As stated above, these parameters are updated during the backpropagation procedure.

**Figure 7:**
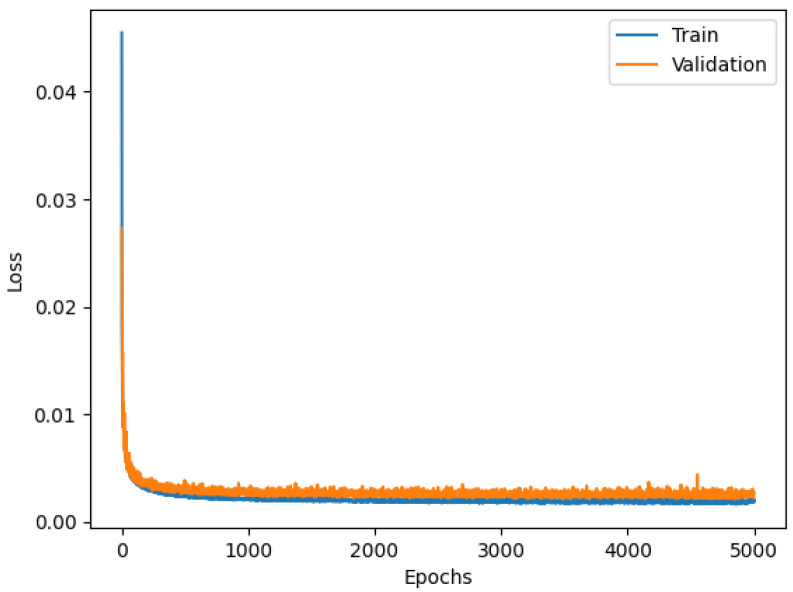
Loss functions of the Dense model.

Both the train and the validation plots are shown in Figure 7. The training curve illustrates how the network is optimizing its parameters during the training phase. The curve illustrates how the network performs on the Test dataset.

The current state of the model can be evaluated at each step of the training algorithm. The training curve related to the Train dataset illustrates the “learning” behavior of the model. The Test dataset is not involved in this training phase. Moreover, the network’s evaluation of the Test dataset provides a clear picture of the inferential capacity of the model. In this specific case, the two curves almost overlap with one another, indicating a proper design of the NN.

### 3.3. Conv1D Model

The second model under consideration is a 1-D Convolutional Neural Network (CNN) [48,49]. CNN models were developed for image classification problems, in which the model learns an internal representation of a two-dimensional input in a process referred to as feature learning. The same process can be harnessed on one-dimensional sequences of data, such as the time sequences of navigation parameters of the considered task. The model learns to extract features from sequences of observations and passes this information to the Dense layer used for the regression task.

The benefit of using CNNs for sequence classification is that they can learn from the raw time series data directly. The model can learn an internal representation of the time series data and ideally achieve comparable performance to model fit using a version of the dataset with engineered features.

**Figure 8:**
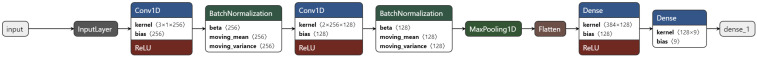
Conv1D model.

The Conv1D model is composed of an input layer with 256 neurons, a hidden layer with 128 neurons, and an output layer with a linear activation function. In addition to the previous model, the batch normalization layer [50] is used after the two convolutive layers, as shown in Figure 8.

The Batch Normalization stabilizes the learning process and drastically reduces the number of training epochs required to train the NN.

In this model, the network is divided into two parts. The convolutive part focuses on feature extraction, trying to obtain as much information as possible from the features. The second part is a DNN dealing with the regression task.

The Max Pooling layer was also considered in the design of this network. A problem with the output feature maps is that they are sensitive to the location of the features in the input. One approach to address this sensitivity is to downsample the feature maps in order to make them more robust to changes in the feature position in the dataset, according to the local translation invariance. Pooling layers provide an approach to downsample feature maps by summarizing the presence of features in patches of the feature map. Two common pooling methods are Average Pooling and Max Pooling, which summarize the average presence of a feature and the most activated presence of a feature, respectively.

Table 3 summarizes the relevant hyperparameters of the exploited Conv1D model together with a brief description of the network architecture.

As can be appreciated, the memory footprint associated with the model parameters is about a quarter of that characterizing the DNN, thus making it feasible for deployment on an edge computing device.

**Figure 9:**
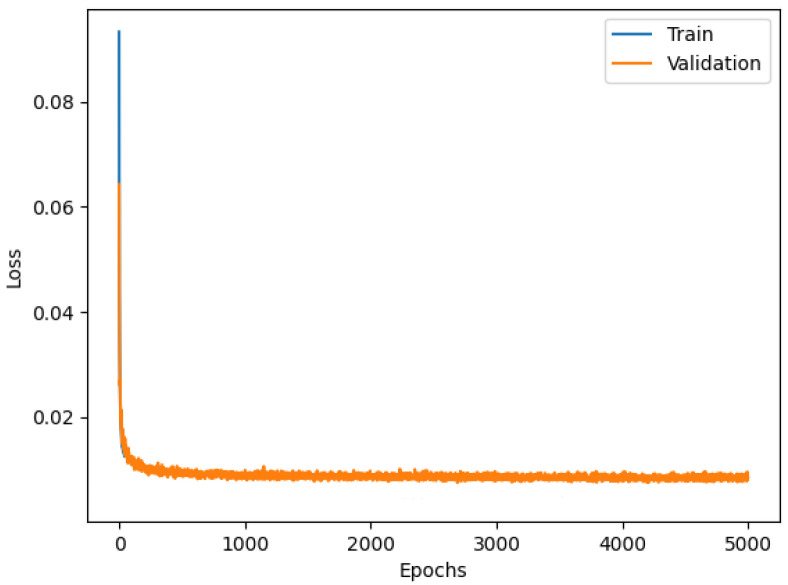
Loss functions of Conv1D model.

The Conv1D model shows a validation curve overlapping the training curve, as shown in Figure 9, an indication of good training of the NN.

### 3.4. LSTM Model

**Figure 10:**
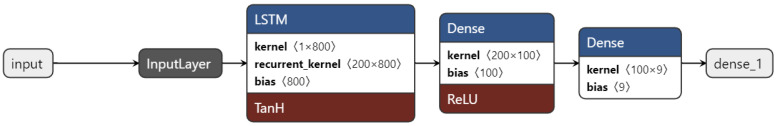
LSTM model.

A Recurring Neural Network (RNN) was considered for the third NN model. In particular, Long Short-Term Memory (LSTM) [51] Network is an advanced, recurrent architecture that allows relevant information to persist by filtering out unnecessary information. LSTM ANN has internal mechanisms called gates that can regulate the flow of information. These gates can learn which data in a sequence is important to keep or throw away. By doing that, it can pass relevant information down the long chain of sequences to make predictions. The exploited LSTM model is composed of an input layer with 200 neurons, a hidden dense layer with 100 neurons, and an output of a dense layer, as shown in Figure 10.

Table 4 shows the most significative hyperparameters related to the LSTM model with a brief description of the network architecture. The number of parameters is 182,698. The memory footprint of this model is located, in terms of bytes, between the dense model (high number of parameters) and the Conv1D model (low number of parameters).

**Figure 11:**
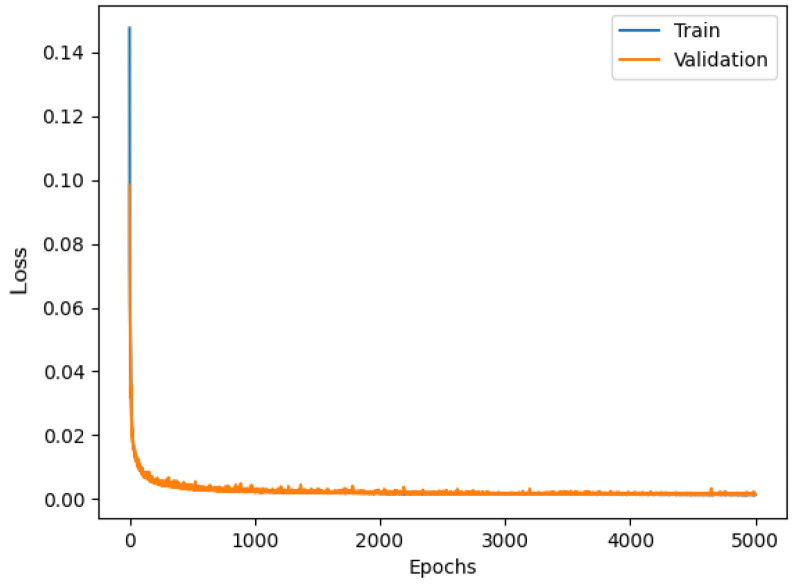
Loss functions of LSTM model.

The LSTM model results in a validation curve almost overlapping the training curve, as shown in Figure 11. It can be concluded that this overlapping is once again a sign of the successful training of the model. Regarding the performance of the LSTM model, the *RMSE* between the predicted values (generated from the network) and the target values (collected from STIM300) is equal to 0.12559. Therefore, both for the *RMSE* and for the impact on the memory footprint, the LSTM model is positioned in the middle between the two models seen above.

### 3.5. Dense Model Optimization through Meta-Heuristic Optimization Techniques

As stated in Section 3.2, the dense NN model was characterized by the worst memory footprint among all the considered models; on the other hand, as it will be shown below, the dense model provided the best performance in terms of *MAE* and *RMSE*. Due to such limitations, research activities were mainly focused on simultaneously optimizing both the prediction performance and the number of dense model parameters. This requirement can be satisfied if optimal hyperparameter values of the DNN models are singled out. This optimization stage was carried out by means of meta-heuristic optimization techniques; in particular, the Particle Swarm Optimization (PSO) [52,53,54] algorithm has been used to finely tune the dense model hyperparameters, thus combining its inherent performance in prediction with a lower memory footprint with respect to the original implementation.

The PSO is able to determine the solution that minimizes a defined cost function within the hyperspace of all the possible solutions. According to our application, the solutions space is given by (i) the number of neurons for each layer, (ii) batch size, and (iii) the learning rate of the DNN, while the cost function takes into account both *MAE* of the predicted navigation quantities and the number of model parameters. PSO will find the optimal solution according to bio-inspired strategies (e.g., bird behavior) by continuously narrowing the search interval around solutions characterized by lower values of the cost function.

**Figure 12:**
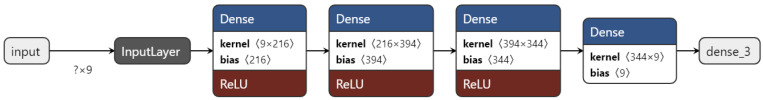
Dense model with Particle Swarm.

As a result of the optimization procedure, the dense NN model was composed of an input layer with 216 neurons, two hidden layers with, respectively, 394 and 344 neurons, and an output layer (Figure 12).

Table 5 shows the most significative hyperparameters related to the optimized Dense model with a brief description of the network architecture.

**Figure 13:**
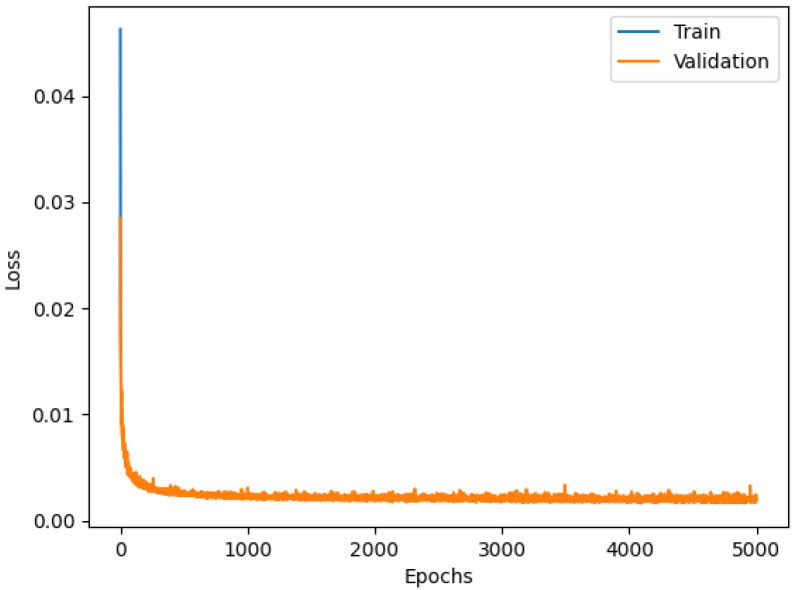
Loss functions of Dense model with Particle Swarm.

The first benefit coming from hyper-parameter tuning is the reduction of the memory footprint compared to the Dense model without parameter optimizations (226,643 parameters, about half of the Dense previous model). The Dense model with PSO shows good training behavior, as shown in Figure 13.

This model overcame the performance, in terms of accuracy, of the first Dense model lowering the *RMSE* from 0.10847 to 0.07783. This last result shows that the Dense architecture trained with the Particle Swarm Optimization algorithm is the best among the analyzed ones.

## 4. Results

This section presents the performance evaluation of four NN models where the results are obtained from the test dataset. Additionally, a comparison is made among the models in terms of error and memory footprint. The error is analyzed through the *RMSE* (Root Mean Squared Error) between the Cube estimates and the predictions generated by the neural network models. The analysis is divided into two parts: *RMSE* between input (Cube) and target (STIM) and *RMSE* between prediction and target.

Table 6 shows the *RMSE* between the Cube and STIM outputs; in particular, the value of the overall *RMSE* between Cube output and STIM output is 5.75871, which is considered as the reference error to be compensated by using ANN models.

### 4.1. Dense Model Performance

The first model tested in this study is the Dense model. The results of the test show a significant reduction in the *RMSE*. A remarkable error reduction can be seen in the entire dataset; in particular, this reduction shown in Table 7 appears to be greater than an order of magnitude. Similarly, the individual features also show an *RMSE* value reduced by at least an order of magnitude.

Table 7 shows the results of the Test dataset to evaluate the inferential capacity of the ANN model: a row of input data (all characterized by the same sample rate) is randomly injected into the NN as input, highlighting how this ANN architecture corrects the Cube estimates.

The model acquires the input and generates the prediction without knowledge of the target. The first line of Table 8 highlights the input data (Cube); the second line reports the predicted data generated by the network; lastly, the third line shows the target data (STIM). It is useful to highlight that the prediction makes significant corrections on some features, such as altitude, roll, and speed components along the x and z-axis.

### 4.2. Conv1D Model Performance

Leveraging this ANN architecture lowers the *RMSE* by about an order of magnitude (Table 7). Similarly, some of the individual features also show an *RMSE* value reduced by at least one order of magnitude. It is useful to highlight that the performance of this architecture is lower than the Dense Network. However, this network has a lower number of parameters and hence a lower memory footprint compared with those of the Dense model. Table 8 shows how the Conv1D model corrects the Cube’s outcome values. It is useful to highlight (see Table 7) that the prediction makes significant corrections on altitude and roll components.

### 4.3. LSTM Model Performance

As shown in Table 7, the outcomes of the LSTM Network model are middle ranking, in terms of performance, between the two models previously analyzed (Dense and Conv1D). Table 9 shows how the network corrects the Cube’s output. This correction allows the network to obtain prediction values close to the target (STIM). It is useful to highlight from the data shown in Table 9 that the prediction makes significant corrections on altitude and speed components along the x-axis and z-axis, which are very close to the target (STIM300).

### 4.4. Performances of the Dense Model with Particle Swarm Optimization Algorithm

The use of the Dense model optimized with Particle Swarm reduces the *RMSE* to a lower value compared to the outcomes of all the other models. In particular, as shown in Tab 7, the global *RMSE* between the prediction generated by this model and the target (STIM) is almost two orders of magnitude lower than the *RMSE* between the input (Cube) and the target (STIM).

After the performance analysis of the individual NN models, this study has focused on a comparison between them.

In the evaluation step, two main aspects were evaluated in detail:

- The reduction of the error between the prediction and the target;
- The NN’s impact on memory footprint;

In order to select the model with better performance, the *RMSE* of each ANN related to the test dataset is shown in Table 10, where the Dense model tuned with Particle Swarm provides the best results. Therefore, in terms of error reduction, the Dense model with PSO could be considered the best-performing one.

In this section, all the discussed ANN architectures are compared to understand which NN model is the most suitable choice for edge devices by evaluating the impact on memory footprint. Table 11 highlights that the Conv1D model is the best candidate for implementation on a microcontroller (uC). The LSTM architecture is the middle ranking, with respect to the optimized Dense model and Conv1D, in terms of the number of parameters. At the same time, the Dense model (without hyperparameters optimization) appears to be less suitable for implementation on microcontrollers.

### 4.5. Trajectories Comparison

In conclusion, in order to have another point of view on the results obtained, in this section, a comparison between the real trajectories (STIM300) with those identified by the Cube and by the predictions of the Dense model with PSO have been evaluated. These results are obtained through the scatter plot of the Latitude and Longitude components.

**Figure 14:**
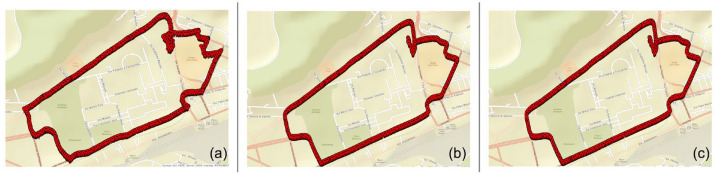
Latitude and longitude estimates: (**a**) Cube, (**b**) Dense NN, (**c**) STIM300.

Figure 14 shows the real path followed during the data acquisition of both the Cube and the STIM. These scatter plots (latitude vs. longitude) represent the trajectories estimated, respectively, by Cube, optimized Dense Net, and STIM300.

**Figure 15:**
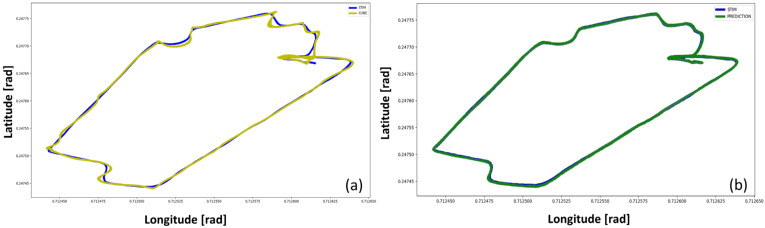
Trajectory estimates comparison: (**a**) target plot vs. input plot, (**b**) target plot vs. prediction plot.

To highlight the good inferential capabilities of the network, the overlapped trajectories plots have been provided in Figure 15. The overlapping of the STIM300 trajectory with both the Cube and the net trajectories better explains the correction made by the NN model on the Cube outcomes.

In Figure 15a, it can be seen that the Cube’s trajectory differs from the target plot in several points. On the other hand, in Figure 15b, the prediction plot (ANN) overlaps the target plot (STIM300) almost perfectly. Therefore, Figure 15b shows the improvements and the soundness of the NN in correcting the trajectory estimated by the Cube.

**Figure 16:**
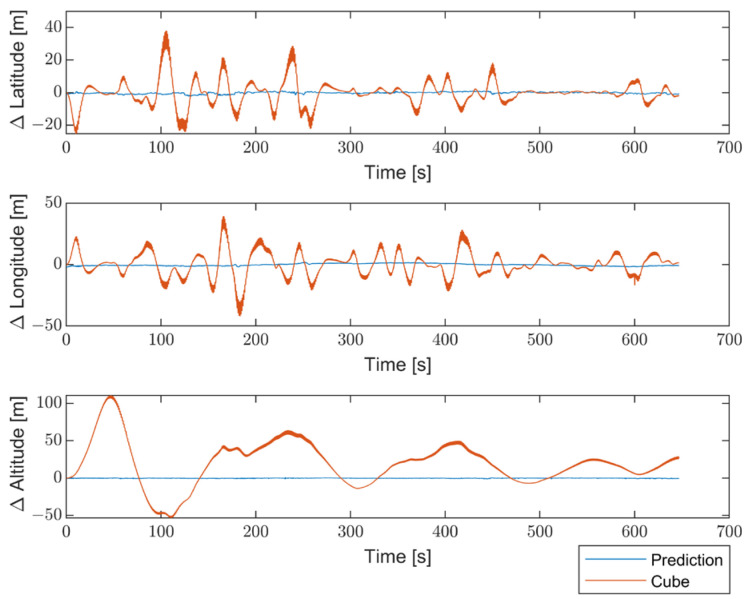
Comparison between the proposed method and the non-optimized Cube in terms of differences in position estimates (ΔLatitude, ΔLongitude, and ΔAltitude) from the Target, in meters.

**Figure 17:**
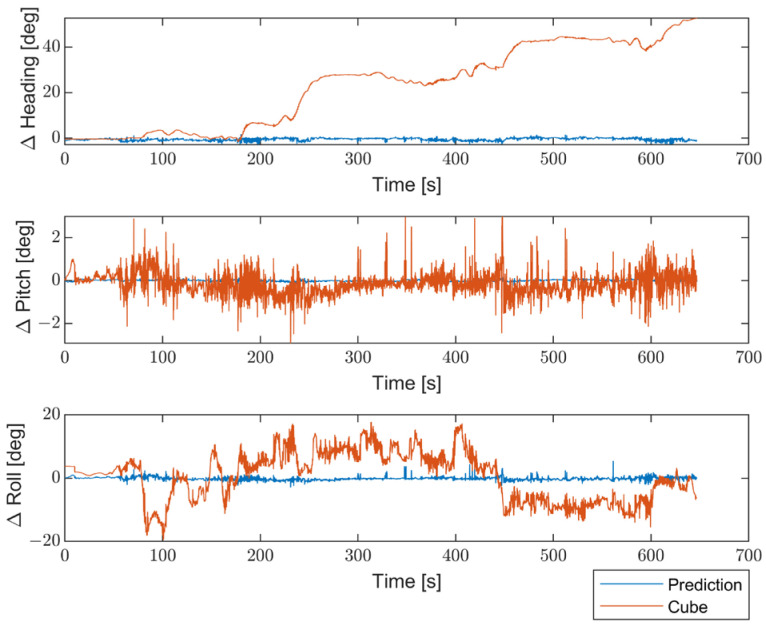
Comparison between the proposed method and the non-optimized Cube in terms of differences in attitude estimates (ΔHeading, ΔPitch, and ΔRoll) from the Target, in degrees.

**Figure 18:**
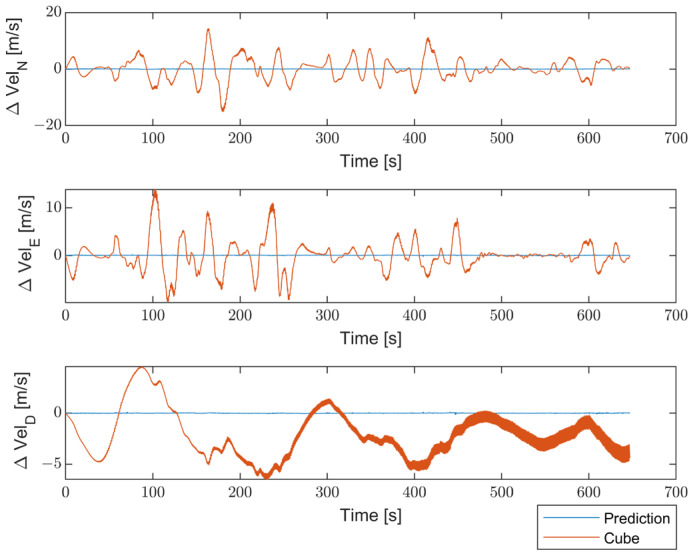
Comparison between the proposed method and the non-optimized Cube in terms of differences in velocity estimates (Δ$Vel_{N}$, Δ$Vel_{E}$, and Δ$Vel_{D}$) from Target, in m/s.

Moreover, to better emphasize the enhancements introduced by the proposed method compared with non-optimized inertial sensors, the results are presented in terms of the differences between the STIM300 (Target) and the predicted values, as well as between the Target and the non-optimized Cube. Figure 16 shows the differences in position estimates (ΔLatitude, ΔLongitude, and ΔAltitude) in meters. Figure 17 exhibits the differences in attitude estimates (ΔHeading, ΔPitch, and ΔRoll) in degrees. Finally, Figure 18 shows the differences in velocity estimates in the NED reference (Δ$Vel_{N}$, Δ$Vel_{E}$, and Δ$Vel_{D}$) in m/s.

The obtained results indicate a significant enhancement in performance for all navigation parameters when compared to the non-optimized Cube, which implies the absence of Allan Variance in the noise estimates for the Kalman filter.

**Figure 19:**
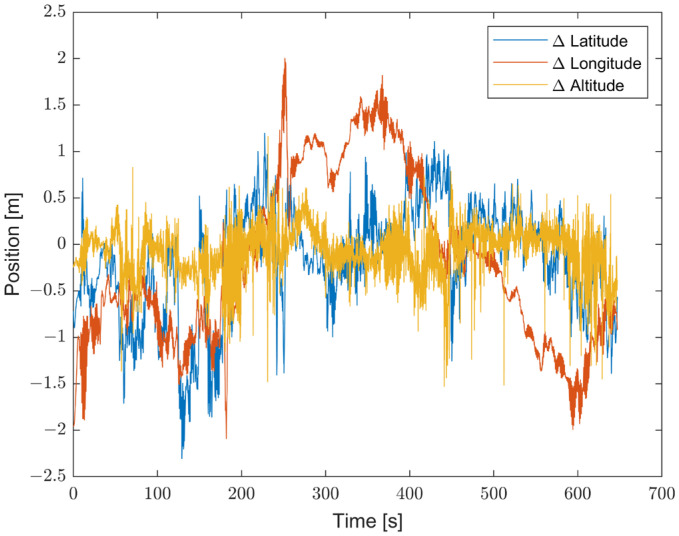
Differences in position estimates (in meters) between the proposed method and Target (STIM300).

**Figure 20:**
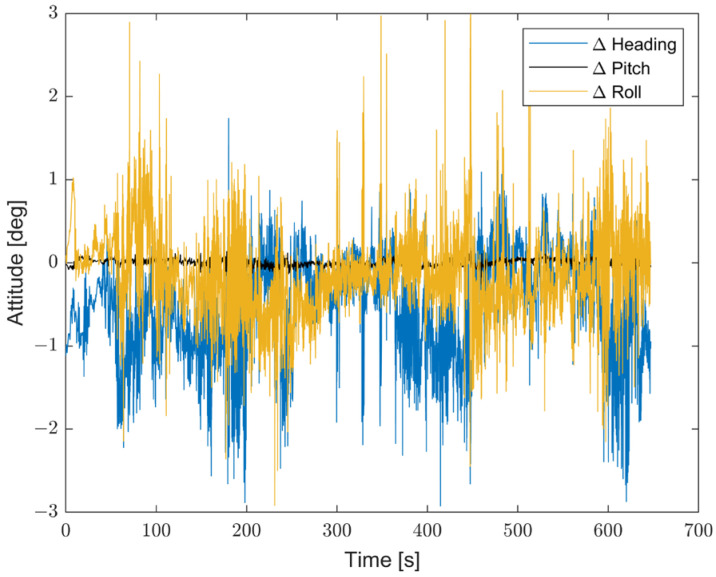
Differences in attitude estimates (in degrees) between the proposed method and Target (STIM300).

**Figure 21:**
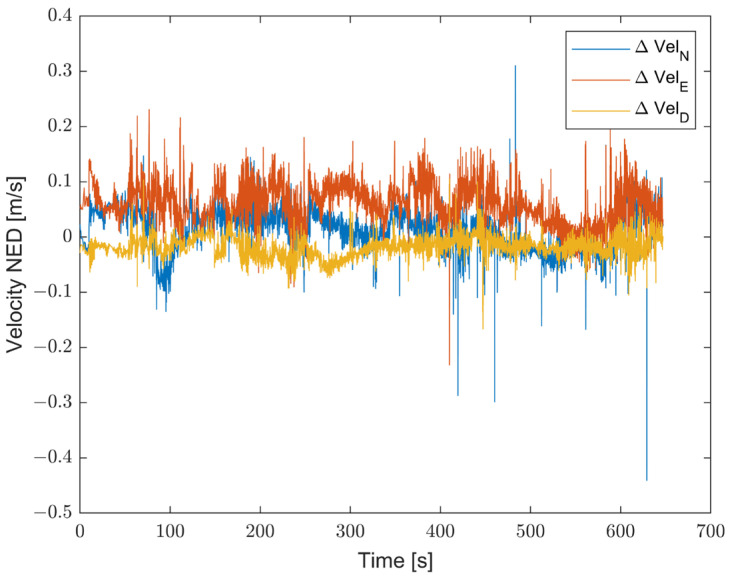
Differences in attitude estimates (in degrees) between the proposed method and Target (STIM300).

Furthermore, the proposed solution shows, as highlighted in Figure 19, Figure 20 and Figure 21, differences for position, attitude, and velocity (NED) on the order of about 1.5 m, 2 degrees, and 0.1 m/s, respectively, with respect to the target, i.e., the STIM300 (tactical-grade IMU).

Finally, with the aim of providing a quantitative parameter to express total system performance, the Performance Factor (PF) is defined according to Equation (3):

(3) $$PF_{i}=\sqrt{\;\left(RMSE_{x}^{2}+RMSE_{y}^{2}+RMSE_{z}^{2}\right)}$$

where *RMSE* is evaluated as differences between the proposed method (or Cube) and the target *i* correspond to the position, attitude, and velocity (NED), while *x*, *y*, and *z* are the latitude, longitude, and altitude values for the position estimates, heading, pitch and roll angles for the attitude estimates and then the velocity estimates (in NED reference frame); the PF results obtained are reported in Table 12 to appreciate better the overall performance achieved.

## 5. Conclusions

The aim of this work has been the analysis, development, and assessment of three ANN models to realize an Inertial Navigation System which uses low-cost sensors with the goal of approaching the accuracies of those obtained through high-end sensors.

The use of NN models was found to be of fundamental importance in improving the performance of a low-cost sensor (SensorTile^TM^) for the realization of a redundant Inertial Measurement Unit (IMU) with high performance.

According to our results, it can be pointed out that the accuracy achieved through the inertial navigation system based on low-cost sensors together with the use of Artificial Neural Network models is comparable to that based on high-end sensors. System capabilities will also be evaluated for Unmanned Aerial Systems (UAS) with the goal of better-assessing altitude prediction, which, although with small variations, was still estimated correctly.

In conclusion, the validation results obtained by adopted models demonstrate that these NNs models have a remarkable prediction capacity.

The choice of using one over another depends on the context of NN applications. For instance, the outcomes of this study have proven that the Conv1D model could be the best option for the implementation of the model on edge devices. On the other hand, in terms of better accuracies, the Dense model with PSO should be considered the best option.

Given the effectiveness of optimization with the Particle Swarm on the Dense model, further studies should be conducted in this way.

In particular, it could be of great interest to optimize the Conv1D and LSTM hyperparameters with PSO, in order to have an overall yardstick on all the NN models developed.

In addition, with the purpose of using NNs in real-time applications, an evaluation of different kinds of microcontrollers should be undertaken to find the best solution for hosting these models.

## Figures and Tables

**Table 1:** Dataset statistics summary.

	Mean	Std	Min	25%	50%	75%	Max
Lat [rad]	0.7125	0.0026	0.7124	0.7125	0.7125	0.7126	0.7126
Lon [rad]	0.2476	0.0028	0.2474	0.2475	0.2476	0.2477	0.2478
Alt [m]	20.6002	14.8431	0.0003	9.0984	18.5825	28.5465	61.5073
Heading [rad]	−0.4692	1.6914	−3.1400	−1.6821	0.0069	0.7879	3.1400
Pitch [rad]	0.0069	0.0998	−0.2341	−0.0696	−0.0095	0.1116	0.2155
Roll [rad]	0.1519	3.0546	−3.1415	−3.0377	2.9960	3.0627	3.1415
VX [m/s]	−0.1573	5.3205	−10.5311	−4.5739	−1.4232	5.3572	10.1318
VY [m/s]	0.1151	5.7372	−12.9115	−3.6694	−0.0372	5.8233	10.4455
VZ [m/s]	−1.4146	1.2136	−4.9909	−2.1293	−1.1552	−0.5929	1.4289

**Table 2:** Hyperparameters of the dense neural network model.

Model	Optimizer: Adam	Loss: Mean Absolute Error	
Training Hyper-Parameters	Batch Size: 185	Learning Rate: 0.001	Epochs: 5000
Layer		Shape	Param
Dense_1		335	3350
Dense_2		479	160,944
Dense_3		579	277,920
Dense out		9	5220
Total params		447,434	
Trainable params		447,434	
Non-trainable params		0	

**Table 3:** Hyperparameters of the adopted Conv1D neural network model.

Model.	Optimizer: Adam	Loss: Mean Absolute Error	
Training Hyper-Parameters	Batch Size: 152	Learning Rate: 0.008	Epochs: 5000
Layer		Shape	Param
Conv1D_1		256	1024
Batch_Normalization_1		256	768
Conv1D_2		128	65,664
Batch_Normalization_2		128	384
Max_Poolling		128	0
Flatten		384	0
Dense_1		128	49,280
Dense_2		9	1161
Total params		118,281	
Trainable params		117,513	
Non-trainable params		768	

**Table 4:** Hyperparameters of the adopted LSTM neural network model.

Model	Optimizer: Adam	Loss: Mean Absolute Error	
Training Hyper-Parameters	Batch Size: 200	Learning Rate: 0.005	Epochs: 5000
Layer		Shape	Param
LSTM		200	161,600
Dense_1		100	20,100
Dense_out		100	909
Total params		182,609	
Trainable params		182,609	
Non-trainable params		0	

**Table 5:** Hyperparameters of the adopted Dense neural network model with PSO.

Model	Optimizer: Adam	Loss: Mean Absolute Error	
Training Hyper-Parameters	Batch Size: 185	Learning Rate: 0.00107	Epochs: 5000
Layer		Shape	Param
Dense_1		216	2160
Dense_2		394	85,498
Dense_3		344	135,880
Dense_out		9	3105
Total params		226,643	
Trainable params		226,643	
Non-trainable params		0	

**Table 6:** The overall *MAE* and *RMSE* between the input and the target were evaluated on the entire dataset (including all the features), and the *RMSE* was calculated for each feature of the dataset.

Parameter	Cube	Dense	Conv1D	LSTM	Opt. Dense
**Lat [m]**	9.8851	1.9361	4.8929	1.9916	1.5458
**Lon [m]**	10.4388	2.8137	0.6659	2.8589	1.5778
**Alt [m]**	35.64075	0.17919	0.66060	0.24496	0.16951
**Heading [rad]**	0.57497	0.04966	0.02606	0.09868	0.00974
**Pitch [rad]**	0.07879	0.00725	0.00587	0.00722	0.00648
**Roll [rad]**	5.40247	0.38673	0.39741	0.41408	0.35528
**Vx [m/s]**	3.85137	0.08497	0.21840	0.09905	0.09356
**Vy [m/s]**	3.28942	0.07638	0.19832	0.07948	0.07568
**Vz [m/s]**	3.10396	0.03012	0.06646	0.02938	0.02800
***MAE***	0.17360	0.00366	0.00548	0.00524	0.00295
**Memory FP**	N/A	447,434	118,281	182,609	226,643
**Navigation** **Parameters *RMSE***	5.75872	0.10847	0.16087	0.12560	0.07784

**Table 7:** Prediction with Dense architecture.

	Latitude [rad]	Longitude [rad]	Altitude [m]	Heading [rad]	Pitch [rad]	Roll [rad]	V(X) [m/s]	V(Y) [m/s]	V(Z) [m/s]
**Input**	0.7125251	0.2477015	41.25674	0.58036	0.05838	3.03860	6.22518	0.91983	4.12185
**Predict**	0.7125225	0.2477014	32.4903	0.48726	0.11589	3.08995	11.67100	1.32938	1.28093
**Target**	0.7125224	0.2477013	32.52912	0.47043	0.11594	3.08365	11.62465	1.34654	1.27595

**Table 8:** Prediction with Conv1D architecture.

	Latitude [rad]	Longitude [rad]	Altitude [m]	Heading [rad]	Pitch [rad]	Roll [rad]	V(X) [m/s]	V(Y) [m/s]	V(Z) [m/s]
**Input**	0.7125251	0.2477015	41.25674	0.58036	0.05838	3.03860	6.22518	0.91983	4.12185
**Predict**	0.7125241	0.2477015	33.28172	0.47928	0.11387	3.06908	11.79970	1.40192	1.29300
**Target**	0.7125224	0.2477013	32.52912	0.47043	0.11594	3.08365	11.62465	1.34654	1.27595

**Table 9:** Prediction with LSTM architecture.

	Latitude [rad]	Longitude [rad]	Altitude [m]	Heading [rad]	Pitch [rad]	Roll [rad]	V(X) [m/s]	V(Y) [m/s]	V(Z) [m/s]
**Input**	0.7125251	0.2477015	41.25674	0.58036	0.05838	3.03860	6.22518	0.91983	4.12185
**Predict**	0.7125233	0.2477015	33.01997	0.48281	0.11419	3.07922	11.55830	1.09890	1.26482
**Target**	0.7125224	0.2477013	32.52912	0.47043	0.11594	3.08365	11.62465	1.34654	1.27595

**Table 10:** *RMSE* between Cube and STIM.

Models	*RMSE*
Dense	0.00366
Conv1D	0.00548
LSTM	0.00524
Dense with PSO	0.00295

**Table 11:** The number of parameters.

Memory Footprint	Parameters
Dense	447,434
Conv1D	117,513
LSTM	182,609
Dense with PSO	226,643

**Table 12:** Performance Factor evaluations.

	PF Position [m]	PF Attitude [deg]	PF Velocity [m/s]
Prediction	1.142	0.893	0.0825
Cube	37.758	90.23	5.895

## Data Availability

Not applicable.
